# Facilitating effect of the hypnotically altered state of consciousness on decision-making in a situation modeling real-life

**DOI:** 10.1192/j.eurpsy.2024.1713

**Published:** 2024-08-27

**Authors:** Z. A. Kovács, S. László, A. Albert, Z. Janka, I. Szendi

**Affiliations:** ^1^Department of Psychiatry; ^2^ Doctoral School of Interdisciplinary Medicine; ^3^Department of Personality, Clinical and Health Psychology, University of Szeged; ^4^Department of Psychiatry, Kiskunhalas Semmelweis Hospital, Szeged, Hungary

## Abstract

**Introduction:**

Numerous studies have shown a link between hypnotic susceptibility, the hypnotically altered state of consciousness, and the intensity of experienced emotions (De Pascalis et al., 1987; De Pascalis, Marucci, & Penna, 1989; Bryant & McConkey, 1989; Crowson, Conroy, & Chester, 1991; Crawford, Kapelis, & Harrison, 1995). One of the most suitable experimental psychological methods for modeling real-life decisional conditions is the Iowa Gambling Task (IGT) (Bechara, Tranel, & Damasio, 2000). Hypnosis has the potential to provide several benefits in decision-making, although there is limited scientific research on the subject.

**Objectives:**

The main goal of this study was to determine if a hypnotically altered state of consciousness could affect decision efficacy in a real-life modeling situation.

**Methods:**

Forty-eight healthy students (including 28 females and 20 males) from the University of Szeged participated in both the delayed punishment and delayed reward versions of the Iowa Gambling Task under alert and hypnotic states.

**Results:**

During the mid-phase of the tasks while in hypnosis, notably higher performance levels were recorded compared to the alert state. In a simulated real-life scenario, the delayed reward had a more pronounced effect on decision-making efficiency than the delayed punishment. It became evident that the efficient decision-making strategy evolved more rapidly under hypnosis than in an alert state.

**Conclusions:**

The hypnotic state of consciousness in an experimental decision situation modeling real life may accelerate the development of somatic markers, leading to earlier correct decision-making.

**Disclosure of Interest:**

None Declared

